# Prognostic Assessment in Patients with Indolent B-Cell Lymphomas

**DOI:** 10.1100/2012/107892

**Published:** 2012-07-31

**Authors:** Luca Arcaini, Sara Rattotti, Manuel Gotti, Stefano Luminari

**Affiliations:** ^1^Division of Hematology, Department of Hematology and Oncology, Fondazione IRCCS Policlinico San Matteo, University of Pavia, 27100 Pavia, Italy; ^2^Division of Oncology, Department of Oncology and Hematology, University of Modena and Reggio Emilia, 41124 Modena, Italy

## Abstract

Follicular lymphoma (FL) is an indolent lymphoma with long median survival. Many studies have been performed to build up prognostic scores potentially useful to identify patients with poorer outcome. In 2004, an international consortium coordinated by the *International Follicular Lymphoma Prognostic Factor project* was established and a new prognostic study was launched (FLIPI2) using progression-free survival (PFS) as main endpoint and integrating all the modern parameters prospectively collected. Low-grade non-Hodgkin lymphomas were once considered as a heterogenous group of lymphomas characterized by an indolent clinical course. Each entity is characterized by unique clinicobiologic features. Some studies have been focused on prognostic factors in single lymphoma subtypes, with the development of specific-entity scores based on retrospective series, for instance splenic marginal zone lymphoma (SMZL). A widely accepted prognostic tool for clinical usage for indolent non-follicular B-cell lymphomas is largely awaited. In this paper we summarized the current evidence regarding prognostic assessment of indolent follicular and non-follicular lymphomas.

## 1. Introduction

Indolent lymphomas represent more than half of malignant lymphomas and include small lymphocytic lymphomas (SLLs), lymphoplasmacytic lymphoma (LPL), follicular lymphomas (FLs), and marginal zone lymphomas (MZLs). Although patients with low-grade lymphoma have an indolent clinical course, patients' prognosis is quite heterogenous among different subtypes and within each of them. So far several studies have investigated prognosis of indolent lymphomas and demonstrated that different demographic features together with clinical and biological factors have a prognostic impact including age, sex, stage, tumor burden, bulky disease, bone marrow involvement, presence of systemic symptoms, performance status, erythrocyte sedimentation rate (ESR), serum lactate dehydrogenase (LDH) level, anemia, thrombocytopenia, and *β*_2_-microglobulin level. The combination of these parameters has allowed the identification of prognostic scores in different types of lymphoma. 

Attempts to define prognosis in low-grade indolent lymphomas begann in the late 70s. Then, when in 1993 the International Prognostic Index (IPI) was defined for aggressive lymphomas [1] it was also applied to low-grade lymphomas leading to conflicting results [2, 3] and the need for a prognostic index specifically designed for follicular as well as for indolent non-follicular lymphomas clearly emerged. 

Specific prognostic scores for follicular lymphoma have been developed with the collection of large multicenter retrospective series, as ILI (Italian Lymphoma Intergroup) score [4] and the Follicular Lymphoma International Prognostic Index (FLIPI) [5]. Recently, a new prognostic score for follicular lymphomas (FLIPI2), based on prospective multicenter web-based collection of data, was developed [6]. Also in SLL, LPL and MZL some attempts were performed that allowed the development of lymphoma-specific prognostic scores.

## 2. Aim of This Paper

This paper describes currently available prognostic tools for patients with indolent lymphoma including follicular lymphoma and non-follicular subtypes. 

## 3. Follicular Lymphoma

FL is the most frequent low-grade lymphoma in western countries, accounting for 25% of all cases [7]. FL is generally considered a long-lasting indolent disorder but survival duration is quite heterogeneous and precise prognostication especially in the monoclonal antibody era is greatly needed [8]. Until now there is no consensus concerning the optimal first-line treatment for FL patients: among possible options patients may be observed without any specific treatment until disease progression or may receive immunotherapy (rituximab alone) or immunochemotherapy (combination of rituximab and cytotoxic chemotherapies). Some cooperative groups such as the *Groupe d'Etude des Lymphomes Folliculaires* (GELF) from France [9] and the *British National Lymphoma Investigation Group* (BNLI) proposed criteria for initiating treatment in FL patients.

More recently some studies have clearly shown a significant improvement in overall survival of FL patients in the last 15years when compared to historical controls [10, 11]. This improvement is mainly related to the introduction of anti-CD20 monoclonal antibodies (MoAbs) in the treatment arsenal. Due to the recent changes in the treatment standards for patients with FL adequate and updated studies are warranted to provide prognostic tools that are really useful in daily practice. 

## 4. Prognostic Factors in Follicular Lymphoma

Prognostic factors in FL reflect different aspects of the disease; some are directly related to the lymphoma biology such as histological features (pattern, grading, and p.i.) or genetic features and tumor microenvironment. Other factors are connected with the tumor spread (stage, tumor burden, bone marrow involvement, symptoms, etc.) or indirect laboratory surrogates (LDH, anemia, and *β*_2_-microglobulin). Other factors are related to disease modifications after treatment (clinical response and minimal residual disease). Finally, some features are not completely related to lymphoma but are more specifically associated with the patient's status (age, performance status, and comorbidity) [12–14].

Regarding the prognostic impact of molecular markers, it has been analysed the prognostic role of secondary cytogenetic changes [15, 16]. Another complex approach is the use of gene expression profiling (GEP) to evaluate prognosis in follicular lymphoma. A seminal study of GEP was performed by the Leukemia Lymphoma Molecular Profiling Project in nearly two thousands patients with untreated FL [17]. Two signatures were identified: the ‘‘immune-response 1” signature, including genes encoding for T-cell markers and genes that are preferentially expressed in macrophages, predicted a favorable outcome and the ‘‘immune-response 2” signature, including genes that are highly expressed in macrophages, dendritic cells, or both, predicted an unfavorable outcome.

Other studies investigated the role of microenvironment (follicular dendritic cells, T cells, histiocytes, and macrophages): results are contradictory, and this could be related to different therapeutic approaches in analysed cohorts of patients [18].

[^18^F] Fluorodeoxyglucose-positron emission tomography (FDG-PET) is a powerful functional imaging tool in staging and response assessment in Hodgkin lymphoma and in diffuse large B-cell lymphoma [19, 20]. Follicular lymphoma is an [^18^F] FDG-avid disease, since more than 90% of patients show a PET-positive disease and sensitivity of staging PET is usually higher than 95% [21–24]. Recent data from a large multicenter clinical trial in advanced FL patients (PRIMA trial) showed that FDG-PET-CT status at the end of immunochemotherapy is strongly predictive of outcome [25]. 

## 5. Prognostic Indexes in Follicular Lymphoma

The International Prognostic Index (IPI) was originally developed for aggressive NHLs but several groups have tested it also in FL patients, confirming that the IPI could discriminate FL patients into subgroups with significantly different survival [3, 26, 27].

In 2004, the Follicular Lymphoma International Prognostic Index (FLIPI) was proposed from the retrospective analysis of more than 4,000 patients with FL treated between 1985 and 1992 [28]. After a multivariate analysis, 5 parameters resulted in being predictive: age > 60 years, serum LDH level > upper limit of normal (UNL), number of nodal areas > 4, and hemoglobin level < 12 g/dL. Three risk groups (low, intermediate, and high) were distinguished (Table 1). The efficacy of FLIPI index has been confirmed in independent series [27, 29, 30].

Interestingly, FLIPI resulted in being predictive of progression-free survival (PFS) in FL patients receiving immunochemotherapy such as rituximab-cyclophosphamide-vincristine-prednisone (R-CVP) [31] or rituximab-cyclophosphamide-doxorubicin-vincristine-prednisone (R-CHOP) [32].

The recent improvement in survival of FL patients makes it difficult to employ OS as endpoint for statistical analysis. In addition, PFS is recommended as the primary endpoint for clinical trials [20]. At the time of the analysis of FLIPI, most of the patients were never treated with immunotherapy, such as rituximab or radioimmunotherapy, and some important clinical and laboratory parameters were not available (e.g., the size of the largest tumor mass and serum *β*_2_-microglobulin levels). For these reasons, in 2004 an international consortium coordinated by the International Follicular Lymphoma Prognostic factor project was established and a new prognostic study was launched (FLIPI2 study) using PFS as main endpoint and integrating all the modern parameters prospectively collected in the univariate and multivariate analyses. The FLIPI2 thus built relies on 5 prognostic parameters: longest diameter of the largest tumor mass > 6 cm, serum *β*_2_-microglobulin level > UNL, bone marrow involvement, hemoglobin ≤ 12 g/L, and age > 60 years [6] (Table2). 

A subsequent study involved 498 patients consecutively diagnosed with FL between 1980 and 2008 in a single institution [33]. 418 had all the parameters needed for FLIPI calculation and 280 patients had all the parameters needed for FLIPI2 calculation. To compare the performance of two predictors in terms of discriminatory power, the Harrell C statistics were used [34]. Applying Harrell C statistics to each parameter of 2 prognostic scores, FLIPI2 produced a more discriminating index compared to FLIPI. 

In addition to FLIPI and FLIPI2, several data are now available suggesting that novel prognostic factors may be relevant for supporting clinical decision in FL patients; among these the study of minimal residual disease and the use of FDG-PET to define the quality of response to systemic treatment are the most promising ones. 

## 6. Indolent Non-Follicular Lymphoma

The REAL classification in 1994 [35], as well as the subsequent WHO classifications in 2001 [36] and 2008 [37], besides the more frequent group of FL, comprised other specific subtypes of low-grade lymphomas, namely small lymphocytic, marginal zone lymphoma (of MALT, nodal and splendid type), and lymphoplasmacytic lymphoma.

A subset of patients with indolent non-follicular lymphomas are frequently managed with a *watch and wait* policy, and treated only at progression. In this regard in 2002 a study focused on and validated previous prognostic criteria for *watch and wait* policy and identified LDH level and number of extranodal sites having prognostic relevance for progression-free survival (PFS) [38]. On the other side, patients with advanced-stage disease are commonly treated with a systemic therapy, ranging from oral alkylators to modern immunochemotherapy approaches. Also in this subgroup a prognostic score able to guide the choice of appropriate therapeutic strategy in individual patients is greatly needed, giving that the few studies aimed to this purpose [2] were based on patients treated before the introduction of the novel monoclonal antibodies and purine analogues.

Moreover, several demographic, clinical, and biological factors demonstrated the same predictive relevance when evaluated in different lymphoma subtype-specific retrospective series [39–42]. 

Some papers have been focused on describing and validating prognostic factors in single lymphoma subtypes, with the development of specific-entity scores based on retrospective series, for instance, splenic marginal zone lymphoma (SMZL). 

In 2006 the *Intergruppo Italiano Linfomi* (IIL) (now *Fondazione Italiana Linfomi*) carried out a study to assess the outcome of splenic marginal zone lymphoma (SMZL) and to identify prognostic factors in 309 patients [39]. Values that maintained a negative influence on lymphoma-specific survival (LSS) in multivariate analysis were hemoglobin less than 12 g/dL, LDH level > UNL, and albumin level less than 3.5 g/dL. Using these 3 variables, 3 prognostic categories emerged: low-risk group (41%) with no adverse factors, intermediate-risk group (34%) with one adverse factor, and high-risk group (25%) with 2 or 3 adverse factors. The 5-year CSS rate was 88% for the low-risk group, 73% for the intermediate-risk group, and 50% for the high-risk group. Recently, the international SMZL Study Group reported a retrospective study on 593 SMZL patients. In the training set, hemoglobin (*P* = 0.003), platelet count (*P* = 0.043), LDH (*P* = 0.011), and extrahilar lymphadenopathy (*P* = 0.020) were the factors independently influencing LSS. Applying specific cut-points low-, intermediate- and high-risk groups with significantly different 5-year LSS of 94%, 78%, and 67%, respectively, were identified. In the validation set the system also separated 3 groups with significantly different 5-year LSS [43].

## 7. New Projects on Prognostication of Indolent Lymphomas

So far, a widely accepted prognostic tool for clinical usage for indolent non-follicular B-cell lymphomas is lacking. Although comprising a heterogeneous group of single rare diseases, with specific biologic and clinical features, low-grade non-follicular lymphomas displayed many common characteristics as indolent behaviour, treatment management and long-lasting survival. 

For these reasons, giving the success of FLIPI2 project for FL, we thought it would be useful to start a new study (*NF10 study*) based on the prospective registration in a relatively short period of time of patients with indolent low-grade non-follicular lymphoma for whom it would be possible to collect an exhaustive set of clinical data and biological information. Moreover, prospective collection of data could eventually allows to collect epidemiological data and build time-dependent prognostic scores, that is, models where patients acquiring additional risk factor at a time subsequent to the diagnosis could shift to the next prognostic level and their follow-ups are splitted in two separate parts and the two parts are treated as different subjects. These models have already been demonstrated to be promising in chronic diseases, such as myeloproliferative neoplasms [44], and could reveal interest in assessing the prognosis at any time during the whole natural history of long-lasting diseases as indolent non-follicular low-grade lymphomas. 

## 8. Conclusions

In conclusion, although FL is generally defined as an indolent lymphoma, patients' outcome is heterogeneous and can be predicted by several prognostic factors and by specific available prognostic models. Studies in molecular biology [45] and PET response [25, 46] identify novel prognostic factors that will represent the basis for future development of risk-adapted therapies.

So far, a widely accepted prognostic tool for clinical usage for indolent non-follicular B-cell lymphomas is largely awaited. For these reasons, giving the success of FLIPI2 project for FL, the *Fondazione Italiana Linfomi* launched a prospective study (*NF10 study*) for defining a new prognostic tool for indolent low-grade non-follicular lymphomas.

## Figures and Tables

**Table 1:** Risk categories according to FLIPI index.

Risk group	Number of factors	Distribution of patients (%)	5-year OS, % (SE)	10-year OS, % (SE)	RR	95% CI
Low	0-1	36	90.6 (1.2)	70.7 (2.7)	1.0	NA
Intermediate	2	37	77.6 (1.6)	50.9 (2.7)	2.3	1.9–2.8
High	≥3	27	52.5 (2.3)	35.5 (2.8)	4.3	3.5–5.3

**Table 2:** Risk categories according to FLIPI2.

Group	Number of factors	Percentage of patients	3-year PFS (%)	5-year PFS (%)	HR (95% CI)
Low	0	20	91	79.5	1.0
Intermediate	1-2	53	69	51	3.19 (2.0–5.15)
High	≥3	27	51	19	5.8 (3.5–9.4)
